# Magnitude and associated factors of low back pain among nurses working at intensive care unit of public hospitals in Amhara region, Ethiopia

**DOI:** 10.1371/journal.pone.0260361

**Published:** 2021-12-02

**Authors:** Bitew Zewudie Tefera, Haymanot Zeleke, Abebe Abate, Haimanot Abebe, Zebene Mekonnen, Yihenew Sewale

**Affiliations:** 1 Department of Nursing, Wolkite University, Wolkite, Ethiopia; 2 Department of Nursing, Debre Markos University, Debre Markos, Ethiopia; 3 Department of Nursing, Debre Birihan University, Debre Birihan, Ethiopia; Sunway University, MALAYSIA

## Abstract

**Background:**

Low back pain is a common public health problem throughout the world with a global prevalence from 28% to 86%. Nurses working in intensive care units are handling people who are critically ill and helpless, which requires more assistance for transferring and handling activities. This possesses a risk for low back pain but little is known about it in Ethiopia. This study aimed to assess the magnitude of low back pain and associated factors among nurses who work at intensive care units in Amhara region public hospitals, North Ethiopia.

**Methods:**

A multi-centered institution-based cross-sectional study was conducted at Amhara region public hospitals from March 1-30, 2020. A simple random sampling technique after proportional allocation was used to select the study participants. Data were collected using a standard modified Nordic musculoskeletal assessment tool. After data were checked for completeness and consistency, it was entered into Epidata version 3.1 and exported to Statistical Package for Social Science software version 26 for analysis. Descriptive statistics were computed. A binary logistic regression model was used to identify factors associated with low back pain. Finally, those variables with a p-value of <0.05 in multivariable analysis were considered statistically significant.

**Result:**

Study was conducted among 412 intensive care unit nurses giving a response rate of 97.6%. The magnitude of low back pain was 313 (76%) [95% CI: (71.6%-79.9%)]. Being female [AOR = 2.674 (1.404, 5.076)], unavailability of assistive device for patient handling [AOR = 2.139 (1.035, 4.410)], lack of training on intensive care [AOR = 2.017 (1.092, 3.943)], lack of regular exercise [AOR = 2.164 (1.164, 4.108)] and job stress [AOR = 3.66 (1.955, 6.498)] were factors significantly associated with low back pain.

**Conclusions:**

In this study the magnitude of low back pain was high. Being female, unavailability of an assistive device for patient handling, lack of training on intensive care, lack of regular exercise and job stress were factors associated with low back pain. Policymakers and concerned bodies should emphasize the accessibility of assistive devices for patient care, provision of training on intensive care, and adaptive working environment for intensive care unit nurses.

## Background

Low back pain (LBP) is pain or discomfort in the spinal area localized between the 12^th^ rib and the inferior gluteal folds with or without radiation to the lower extremities [1]. It is a condition commonly encountered in clinical areas affecting gluteal folds and lower rib cages [2]. It is neither a disease nor a diagnostic entity, but it is a pain of variable duration in the lower back area in response to internal and external stimuli [3]. Nurses are usually affected by low back pain due to their working condition which involves lifting and transporting patients or equipment in a difficult environment, especially in developing countries where assistive devices are not available [4]. The intensive care unit (ICU) is a special ward in the hospital in which critically ill patients are provided with comprehensive, accurate, and ongoing care. Nurses working in this ward play a crucial role in caring for critically ill patients [5].

Low back pain was the most commonly reported Work-Related Musculoskeletal Disorder among Intensive Care Unit (ICU) Nurses in China (80.1%) [6] and Korea (90.3%) [7]. Similarly, in Turkey, the prevalence of musculoskeletal disorder symptoms among nurses was 79% [8]. The prevalence of LBP among nurses in Africa was found to be 44.1%-82.7% [9] and 45.8–70.9% in Ethiopia [10].

Previous studies identified that being female, married, older age, smoking, lack of regular exercise, being overweight, involved in works requiring frequent twisting and bending, prolonged standing at the workplace, inadequate staff, and heavy weight lifting were factors associated with the experience of low back pain [4, 11]. Low back pain has numerous impacts on nurses, which includes time off work, increased risk of chronicity, as well as increased personal and medical cost [12]. It also leads to impaired professional function and decreased quality of care provided to the patient, negatively affecting the health of the client [13].

Regular exercise to enhance the strength of abdominal and lower back muscles, using proper body mechanics while lifting patient, having rest intervals in occupational duties that require bending for long hours, and maintaining healthy living conditions by avoiding smoking and reducing excessive body weight are crucial for reducing the incidence of low back pain [14]. A multi-dimensional approach like training in intensive care, providing a manual for health care workers, encouraging and sensitizing nurses about safe handling of patients is also vital to prevent low back pain [15].

Even if the problem is huge, there are limited evidence on its magnitude and contributing factors in intensive care unit nurses in Ethiopia. Some previous studies conducted in Ethiopia by including all health care professionals, working in all units may undermine the real prevalence of low back pain among ICU nurses. Nurses who work in the intensive care unit frequently complained of low back pain and asked to have sick leave that needed to be identified with evidence.

The finding of this study revealed data on the magnitude and associated factors of low back pain among intensive care unit nurses in Amhara region public hospitals. Identifying factors associated with low back pain is an important input for policymakers and hospital administrative bodies to plan and implement preventive measures aimed at reducing the problem and associated decreased quality of nursing care. It also helps nurses to modify the behaviors that predispose them to low back pain to avoid unnecessary personal and medical costs. Therefore, this study aimed to assess the magnitude of low back pain and associated factors among nurses who work at intensive care units in Amhara region public hospitals, North Ethiopia.

## Methods

### Study design, setting, and sample size

An institutional-based cross-sectional study was conducted from March 1-30, 2020 in Amhara region public hospitals. Amhara region is one of the ten regional states of Ethiopia with the capital city of Bahirdar, located 565 km away from Addis Ababa, the capital city of Ethiopia with a total population of 22,000,000. There are a total of 82 public hospitals in the region, of which 73 were primary, 3 were general and 6 were referral hospitals. Only general and referral hospitals provide intensive care services for the catchment population.

The required sample size (n) for the first objective was calculated by using single population proportion formula, assuming the prevalence (p) of low back pain as 50% since there were no previous similar studies in the Ethiopia context, 95% confidence interval, 5% margin of error (d) and 10% for possible non-response rate as follows;

$n=\frac{z_{\frac{a}{2}}^{2}(p(q)}{d^{2}}=\frac{1.96^{2}(0.5(1-0.5)}{0.05^{2}}=384$ and by adding 10% for possible non-response rate it becomes **422**. The minimum required sample size for the second objective was also calculated using epi-info software by considering factors associated with low back pain in other studies, 80% power, 1:1 ratio of exposed and non exposed, 95% confidence interval, 5% margin of error, and adding 10% for possible non-response rate. Finally, by taking the maximum value, the final required sample size for this study was **422**.

### Sampling procedure

There was a total of 82 public hospitals in the Amhara region; of those, 73 were primary, 3 were general and 6 were referral hospitals. All general and referral hospitals having intensive care units were included in the study. The nine included public hospitals with their respective number of nurses currently working in intensive care units were:- Debre Markos referral hospital (53), Felege Hiwot comprehensive specialized hospital (65), Tibebe Gion comprehensive hospital (57), University Of Gonder comprehensive specialized hospital (60), Dessie referral hospital (58), Debre Birihan referral hospital (58), Enjibara general hospital (49), Debre Tabor general hospital (30) and Wollo general hospital (52).

**Fig 1:**
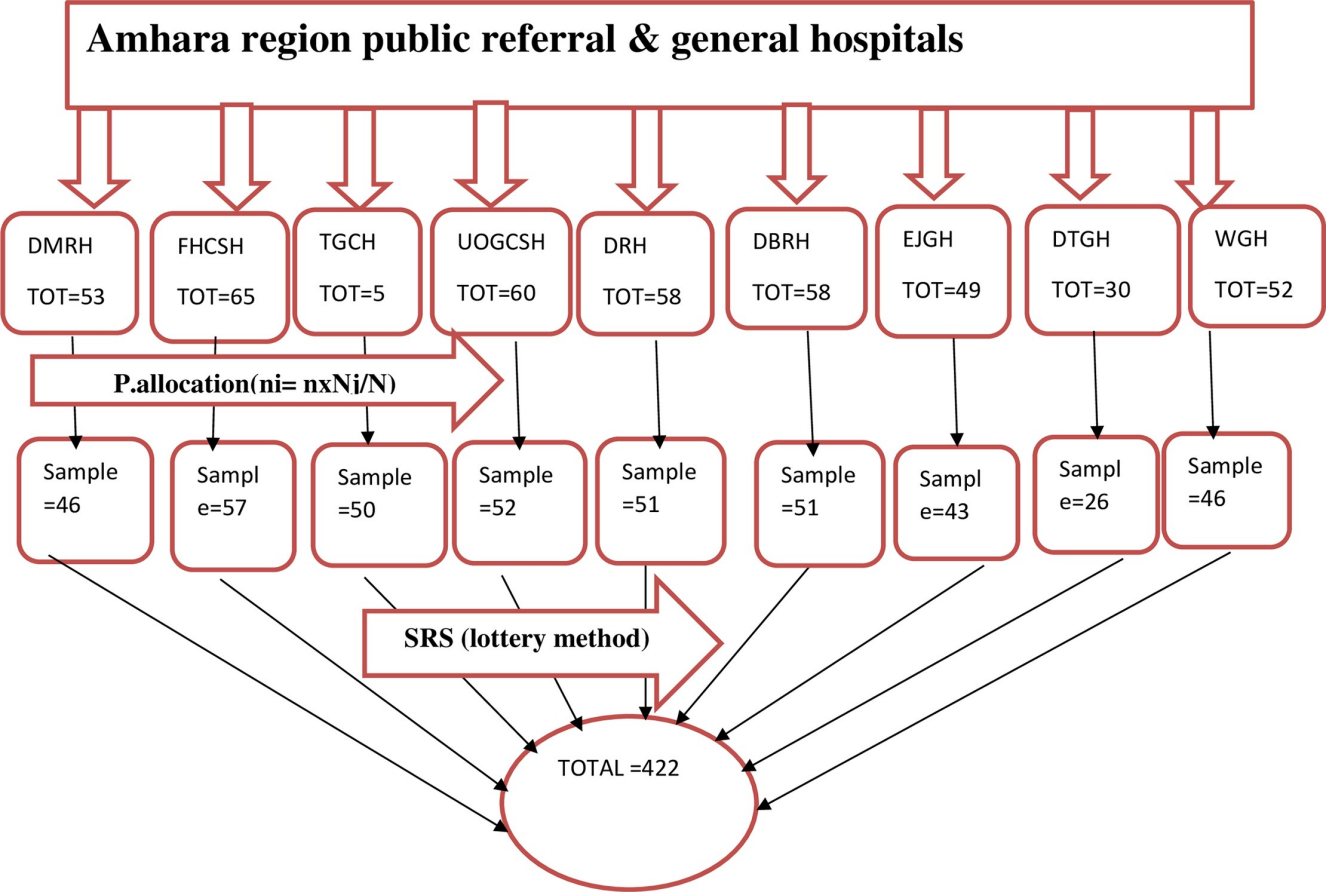
Sampling technique to select study participants among ICU nurses in public referral and general hospitals of Amhara Region, Ethiopia, 2020.

Simple random sampling techniques with the lottery method after proportional allocation were used to select the study participants (n = 422) from each hospital **(Fig 1)**.

### Data collection tool and procedure

Data were collected using a self-administered structured questionnaire adapted from Standard Nordic Questionnaire to assess Musculoskeletal Disorders (MSDs) [16]. The questionnaire had five sections; Socio-demographic characteristics questions containing six items, Low back pain characteristics questions containing six items; and pain intensity was measured by numeric pain rating scale, Organizational related factors questions containing eight items, Work-related assessing questions containing eight items, Individual factor assessing questions containing seven items and job stress was assessed by workplace stress scale containing eight items each rated as 1(never), 2(rarely), 3(sometimes),4(often),5(very often) and A nurse who scores mean and above (> = 21) of workplace stress scale was considered as having job stress. Data were collected by nine nurses and three supervisors after one-day training was given. One nurse for each selected hospital was assigned to collect data.

### Inclusion and exclusion criteria

All nurses currently working in the ICU of Amhara region public hospitals were included in this study of those nurses who had low back pain before joining the ICU ward, nurses who were pregnant, and nurses who joined the intensive care unit less than twelve months were excluded.

### Operational definitions

#### Low back pain

Any pain felt in the low back region (localized between the 12^th^ rib and the inferior gluteal folds) for at least one-day duration in the last twelve months [10, 17].

#### Mild LBP

Pain intensity on numeric scale score of 1–3 [10].

#### Moderate LBP

Pain intensity on numeric scale score of 4–6 [10].

#### Severe LBP

Pain intensity on numeric scale score of 7–10 [10].

#### Job stress

A nurse who scores the workplace stress scale of 21 or above [18].

#### Regular exercise

Performing physical activity three days/week for 20–30 minutes duration [19].

### Data quality control

A pilot test was done on 5% (21) of the sample at Ras Desta Damitew memorial hospital, Addis Ababa to check the reliability of the questionnaire. One day of training was given for the data collectors and supervisors before the actual data collection. Emphasis was given on the significance and the appropriate meanings of each question as well as how to explain it to the participants in an understandable manner. Daily basis review and checkups were done on each questionnaire for completeness, accuracy, and consistency of the collected data. The reliability of the tool to assess job stress was checked by Cronbach’s alpha value (0.86).

### Data processing and analysis

After data were checked for completeness and consistency, it was entered using Epi-data software version 3.1 and exported to SPSS software version 26 for analysis. Descriptive statistics were computed and presented using text, tables, and graphs. Binary logistic regression analysis was used to identify factors associated with low back pain. Variance inflation factors were used to check for the presence of multicollinearity. Model goodness of fit was tested by using Hosmer-Lemeshow statistic. All variables with P<0.25 in the bivariate analysis were included in the final model of multivariable analysis to control all possible confounders. The degree of association between dependent and each independent variable was assessed using an adjusted odds ratio with 95% CI and variables that have a p-value of <0.05 were considered statistically significant.

### Ethics consideration

Ethical approval was obtained from Debre Markos University ethical review board. Informed and voluntary written consent was obtained from each study participant. Confidentiality of personal information was assured through coding and aggregate reporting. All COVID-19 preventive measures were implemented throughout the data collection period.

## Results

### Socio-demographic characteristics of the participants

From 422 total sample size, 412 intensive care unit nurses participated in the study giving a response rate of 97.6%. The mean age of participants was 30 years with a standard deviation of ±3.56. The majority of study participants were between 30–39 years old (89.3%) and married 233 (56.6%) **(Table 1)**.

**Table 1:** Socio-demographic characteristics of intensive care unit nurses in Amhara Region public hospitals, Amhara, Ethiopia, 2020 (N = 412).

Variable	Category	Frequency	Percent
Age	20–29	10	2.4%
	30–39	368	89.3%
	> = 40	34	8.3%
Sex	Female	217	52.7%
	Male	195	47.3%
Ethnicity	Amhara	349	84.7%
	Tigre	22	5.3%
	Oromo	41	10.0%
Religion	Orthodox	297	72.1%
	Muslim	57	13.8%
	Protestant	53	12.9%
	Others (Catholic)	5	1.2%
Educational status	Diploma	54	13.2%
	Degree	328	79.6%
	Masters and above	30	7.2%

### Organizational related factors

Almost half of the respondents (49.8%) were from referral hospitals. Out of 412 study participants, 341 (82.8%) of them were working in shifts (day and night alternatively) and fifty-five percent of the respondents work in different units with year-based shifting schedules. Regarding staff adequacy, 294 (71.4%) of the respondents reported that there was no adequate staff for assistance during patient transferring and handling activities. Two-thirds (66%) of participants reported that there were assistive devices in their working unit like a wheelchair, transferring bed, etc. for patient handling activities. The majority 343 (83.3%) of the study participants reported that there was no special training center in their working hospital.

### Work-related factors

The majority 349 (84.7%), of the study participants, performed repetitive tasks requiring frequent bending and twisting in their working unit. About 314 (76.2%) of respondents lift heavyweight (>10kg) manually in their unit and position patients frequently. About 70% of participants were standing long (>1 hour) while performing nursing procedures and 281 (68.2%) of them worked while physically fatigued.

### Individual and psychosocial factors

**Fig 2:**
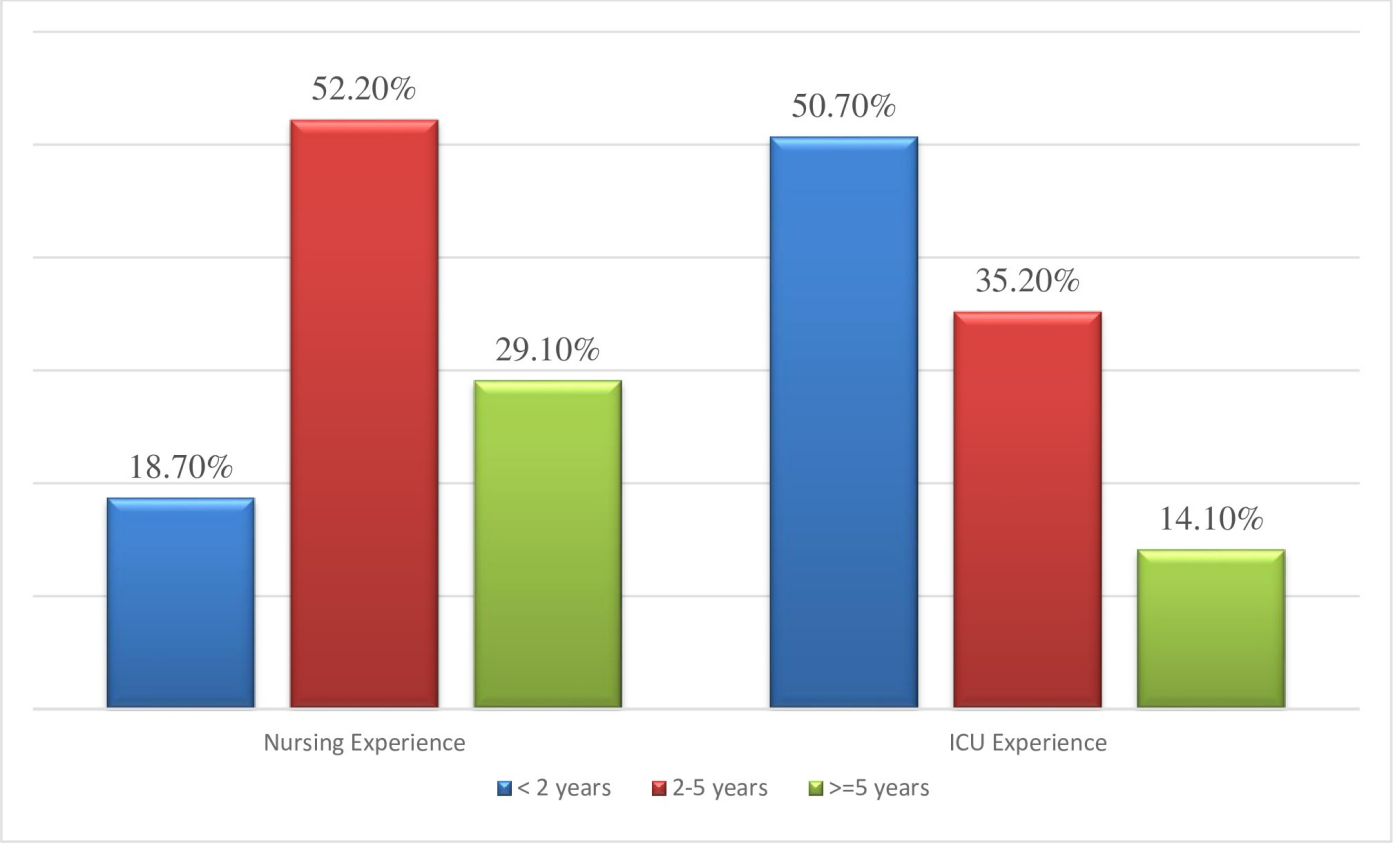
Working experience of nurses in intensive care units of Amhara region public hospitals, Amhara, Ethiopia. 2020.

More than half 215 (52.2%) of study participants have 2 to 5 years of working experience in nursing **(Fig 2)**. The majority of the respondents, 348 (84.5%) had a normal body mass index, and above half (56.3%) of them had no habit of doing regular exercise **(Table 2)**.

**Table 2:** Individual and psychosocial factors of low back pain among ICU nurses in Amhara region public hospitals, Amhara, Ethiopia 2020.

Variable	Category	Frequency	Percent
Current ICU working unit	Adult ICU	193	46.8%
	Neonatal ICU	219	53.2%
Training on ICU	Yes	215	52.2%
	No	197	47.8%
Body mass index (BMI)	<18.5	61	14.8%
	18.5–24.9	348	84.5%
	>25	3	0.7%
How long do you sleep per day	<8hrs	287	69.7%
	≥8hrs	125	30.3%
Job stress	Yes	257	62.4%
	No	155	37.6%

### The magnitude of low back pain and related characteristics

**Fig 3:**
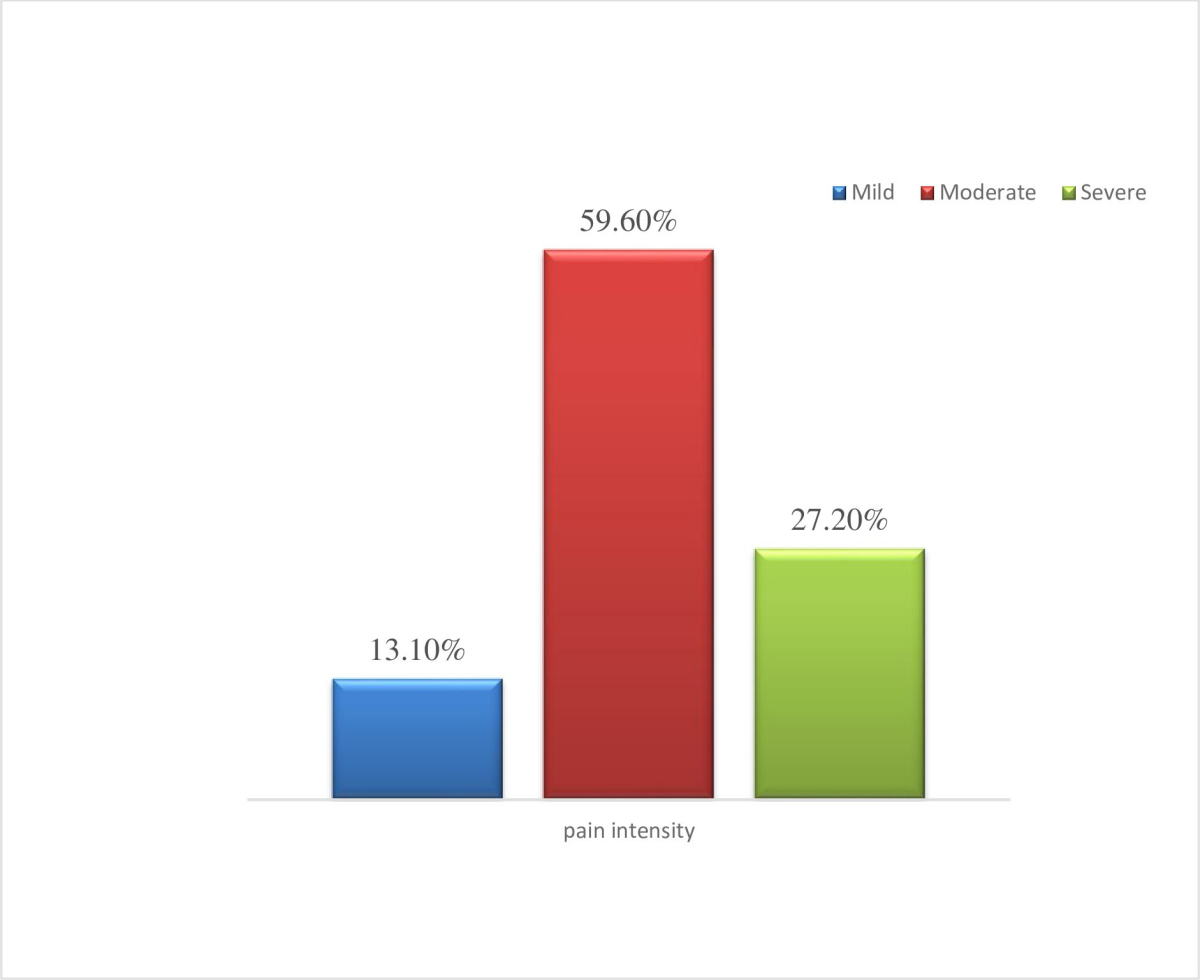
Low back pain intensity level of intensive care unit nurses in Amhara region public hospitals, Amhara, Ethiopia, 2020.

Among the total study participants, 313 (76%) of them experienced low back pain for at least one-day duration in the last 12 months. The majority of them had experienced low back pain levels of moderate-intensity **(Fig 3)**. From those participants who experienced LBP 87 (27.8%) of them had lower extremities radiated pain and 183 (58.5%) of them used antipain medications. Almost one-fourth (24.3%) of study participants with low back pain thought to change their job and 111 (35.5%) of them were absent from work due to low back pain.

### Factors associated with low back pain

In bivariable logistic regression analysis factors such as; sex, age, marital status, work shift, presence of adequate staff, availability of assistive devices, training on intensive care, frequent bending or twisting, manual heavy weight lifting, frequent positioning, and transferring of patients, working when physically fatigue, performing repetitive tasks, long-standing (>1hr), regular exercise, alcohol use, year of experience and job stress were associated with low back pain at p-value <0.25. In multivariable logistic regression analysis; sex, availability of the assistive device, special training on intensive care, regular exercise, and stress were significantly associated with low back pain.

Females were 2.674 times [AOR = 2.674 (1.404, 5.076)] more likely to experience low back pain than males and those nurses working in intensive care units without an assistive device for patient handling in their hospital were 2 times[AOR = 2.139 (1.035, 4.410)] more likely to develop low back pain than those who had assistive devices. The odds of experiencing low back pain were almost two times [AOR = 2.017 (1.092,3.943)] higher among nurses who didn’t take special training on intensive care than the counterpart. Intensive care unit nurses who didn’t perform regular exercise were nearly 2 times [AOR = 2.164 (1.164,4.108)] at risk for experiencing low back pain than those with a habit of regular exercise. Job stress has increased the likelihood of experiencing low back pain by 3.6 times [AOR = 3.66 (1.955, 6.498)] as compared to those who didn’t have job stress **(Table 3)**.

**Table 3:** Multivariable logistic regression analysis of factors associated with LBP among ICU nurses working at Amhara region public referral and general hospitals, 2020 (N = 382).

Variables	Categories	LBP		95% confidence interval		P-value
		Yes	No	COR	AOR	
Sex	Female	183	34	2.691(1.679,4.314)	2.674(1.407, 5.081)	.003*
	Male	130	65	1		
Regular exercise	No	200	32	3.706(2.293,5.990)	2.164 (1.151, 4.067)	.017*
	Yes	113	67	1		
Job stress	Yes	223	34	4.737(2.926,7.668)	3.666 (2.000, 6.720)	.000*
	No	90	65	1		
Assistive devices availability	No	124	16	3.403(1.903,6.085)	2.319 (1.111, 4.841)	.025*
	Yes	189	83	1		
Training on ICU	No	169	28	2.976(1.822,4.861)	2.017 (1.055, 3.856)	.034*
	Yes	144	71	1		

**Abbreviations: COR**: crude odds ratio; **AOR**: adjusted odds ratio, **LBP**: low back pain, **ICU**: Intensive care unit.

* significant variable.

Independent variables significantly associated with the outcome variable are sex, regular exercise, job stress, assistive device availability, and training in ICU.

After performing binary logistic regression analysis and identifying significant variables at the binary level; we performed multivariable logistic regression analysis and the above variables were significantly associated with the outcome variable(LBP).

## Discussion

Low back pain is a major cause of disability that affects the quality of life as well as work performance. The high prevalence of low back pain in intensive care unit nurses negatively affect the quality of care in nursing, since patients in the ICU need nurses’ assistance every minute of their life.

In this study, the magnitude of low back pain was 76% [95% CI: (71.6%-79.9%)]. This is in line with studies conducted in Nigeria (73.5%) [4], Egypt (79%) [11], and Rwanda 78% [20]. This might be due to a similar operational definition of low back pain, study design, and setting.

However, the prevalence of low back pain in this study was lower than different studies conducted in China (80%) [6] and Taiwan (82%) [21]. The discrepancy might be due to differences in pain reporting culture, lifestyle, and socio-demographic characteristics of study participants. Asians expect an extravagant display of emotion in the presence of pain, but African people value stoicism, restraint, and playing down the pain [22, 23]. In addition to this, African individuals considered mild pain as normal and may not report as having pain.

On the other hand, it was higher than a study conducted in Saudi Arabia (51.2%) [24], Malaysia (63.1%), Brazil (67%), Bahirdar, Ethiopia (64.07%) [9], Wollega (63.3%) [18] and Addis Ababa, Ethiopia (45.8%) [10]. The difference might be due to, previous studies include all nurses working in all units, which results in a difference in the assigned task in which they are responsible; where ICU nurses are assigned with critically ill patients needing close follow-up that increase the prevalence of low back pain in the current study.

In this study, females were 2.67 times more likely to develop low back pain than males. This finding is supported by studies conducted in Uganda [25], South Africa [26], and Nigeria [27].

This is due to physiologically low disk space in females that predispose to low back pain and as age increases in females’ estrogen level decreases and collagen wasting occurs, as a result, females might experience low back pain more than males [28]. The physical stress of child-rearing and perimenopausal abdominal weight gain may also contribute to the occurrence of low back pain in females [29].

In this study, ICU nurses who didn’t perform regular exercise were 2.16 times more likely to experience low back pain when compared with those nurses who perform regular exercise. This is in line with studies conducted at Adama hospital medical college staff and turkey Istanbul ICU nurses [17, 30]. The possible justification could be regular exercise improves physical fitness, which prevents easy fatigability of back muscles that reduce the incidence of low back pain [31]. The other possible reason is, those nurses who perform regular exercise can acquire normal body weight, which indirectly prevents low back pain by reducing their weight; since most studies suggested that overweight nurses are more at risk for low back pain when compared with nurses who have normal body weight [11]. This indicates a need to have an exercise center in the health care institutions for health care workers especially for nurses working in an intensive care setting to improve their physical fitness during their leisure times.

In this study, those ICU nurses who had job stress were 3.66 times more likely to experience low back pain when compared with those who didn’t have job stress. This finding is supported by a study conducted at Addis Ababa, Ethiopia among nurses [10]. The possible justification could be when ICU nurses become stressed at work by different assigned tasks, they become physically fatigued and fatigue negatively influences pain receptors by increasing sensation, leading to low back pain. This implies that the working environment should be pleasant, safe, have adequate rest time, and create a sense of team spirit within health care workers.

Lack of special training in intensive care was also the other factor that was significantly associated with the experience of low back pain among ICU nurses. The odds of experiencing LBP in this study were almost doubled in those ICU nurses who didn’t take training on intensive care when compared with those who have taken the training. This study was supported by a study conducted in Wollega [18]. It is also strengthened by an interventional study which was conducted in turkey, that showed health care staff who engaged in training on patient transferring and lifting perform six out of seven behaviors better than they did previously [32]. Even though the exact relationship between taking training and low back pain was not established, training increases awareness of nurses towards preventive measures of low back pain in the workplace that leads to behavioral change and maintenance of workplace safety cultures [18]. This implies that training should be provided for nurses working in the intensive care unit on patient handling and transferring, as well as body mechanics to reduce the burden of low back pain and associated poor quality of care.

In the current study, ICU nurses who didn’t have assistive devices (wheelchair, lift, transfer beds, automated beds…) for patient handling were 2.3 times more likely to experience low back pain when compared with ICU nurses who had an assistive device. This finding is supported by cross-sectional studies conducted in Gaziantep Turkey [33] and Nigeria [31]. The possible justification could be, when ICU nurses use available assistive devices for patient handling activity they can easily perform their activity without fatigability and the workload on their body, especially on their lower back, decreased as a result of the occurrence of low back pain will be reduced.

## Conclusion

The prevalence of low back pain among intensive care unit nurses in the Amhara region was found to be high. Being female, unavailability of an assistive device for patient handling, lack of training in intensive care, lack of regular exercise, and job stress were factors significantly associated with low back pain. Health policymakers and hospital administrative bodies should emphasize the accessibility of assistive devices for patient care, provision of training on intensive care, and adaptive working environment for intensive care unit nurses. Research evidence using prospective cohort studies is also recommended to establish cause and effect relationships.

## Implication for nursing clinical practice

Based on the findings of the study, the presence of an assistive device is crucial for ICU nurses to reduce the occurrence of LBP and to perform their nursing care. it is also important for nurses to have regular physical exercise to prevent developing LBP and to perform their nursing care effectively. Giving ICU training for nurses who are working in ICU is essential for the clinical practice of nurses. Since training enables nurses to use appropriate patient transferring techniques and the occurrence of LBP will also be reduced as a result in those individuals. Recruiting or shifting of adequate nurses for ICU to reduce or overcome the workload of nurses working in ICU is recommended to improve nursing clinical practice.

## Limitation of the study

Since it was a cross-sectional study it may not show cause and effect relationship and may have recall bias.

## Supporting information

S1 File The raw data supporting findings of the article. (SAV)

S2 File Data collection tool for the study. (DOCX)

## Abbreviations

AOR: Adjusted Odds Ratio
BMI: Body Mass Index
ICU: Intensive Care Unit
LBP: Low Back Pain
MSD: Musculo-Skeletal Disorder
SPSS: Statistical Package for Social Sciences
WRMSDs: Work-Related Musculo-Skeletal Disorders
