# Supplementary material for: Magnitude and associated factors of low back pain among nurses working at intensive care unit of public hospitals in Amhara region, Ethiopia
Source: PLoS One. 2021 Dec 2;16(12):e0260361. doi: 10.1371/journal.pone.0260361 (PMC8639077; doi:10.1371/journal.pone.0260361)
Supplement: S2 File — (DOCX) [file pone.0260361.s002.docx]

***Questionnaire******s for respondents***

| **Questions** | **Categories** |
| --- | --- |
| ***Part one: Socio-demographic Characteristics*** | |
| Age of respondent? | ------------------ years |
| Sex of respondent? | 1. Female 2. Male |
| What is your ethnicity | 1. Amhara 2. Tigre  3. Oromo 4. others specify ­­---------- |
| What is your religion | 1. orthodox 2. Muslim  3. protestant 4. others specify_____ |
| What is your marital status? | 1. Married 2. Single  3. Divorced 4. Widowed |
| What is the highest nursing educational level you completed? | 1. Diploma 2. Degree  3. Masters/above |

| ***Part two: -* Low Back Pain (LBP) status** | |
| --- | --- |
| Have you had low back pain in the last 12months since you were working as ICU nurse? | 1. yes 2. no |
| Did your pain radiate to your extremities | 1. yes 2. No |
| Rate your average pain intensity by circling one number that best describes your LBP. Considering that 1= mild pain,5=moderate and 10 =severe pain. | **0 1 2 3 4 5 6 7 8 9 10** |
| How often you feel pain on your low back? | 1. Infrequent (<3 days/ weak)  2. Frequent (3-5 days/ weak)  3. Daily pain (6-7 days/ weak) |
| Did you use any medication for your low back pain? | 1. yes  2. no |
| If yes which Medications, have you taken? | 1. Pain Killer 2. Muscle Relaxant 3. Narcotics 4. Other specify |
| Have you ever thought to change your job because of low back pain? | 1. yes  2. no |
| Have you ever been absent from work due to your low back pain? | 1. yes  2. no |
| **Part Three: Organizational factors** | |
| How is your ICU work shift status? | 1. Permanently  2. Shift worker |
| For how long is you working shift? | 1. Every 3 month  2. Every 6 month  3. Yearly  4. Other/ Specify-------------- |
| Is there adequate staff (at least 6) for assistance while you transferring a patient? | 1. yes  2. no |
| Are there assistive devices for patient handling activities? (lift, wheelchair, transfer bed, automated bed, ) | 1. yes  2. no |
| Are there available back ergonomics training center in your hospital | 1. yes  2. no |
| Do you give medication frequently in your unit | 1. yes 2. no |
| Do you bend /twist while working in your unit | 1. yes 2. no |
| Have you lift manually heavy weight >10kg in your unit | 1. yes 2. no |
| Do you frequently position patient on your bed | 1. yes 2. no |
| Do you work while you are physically fatigue | 1. yes 2. no |
| Have you performed repetitive tasks | 1. yes 2. no |
| Do you stand >1hr per day in your unit while doing nursing procedures | 1. yes 2. no |
| **Part five: -Individual and psychosocial Factors** | |
| How long have you worked as a nurse? | ----------------------years |
| How long have you worked in ICU? | ----------------------years |
| Which ICU are you currently working in? | 1. Adult ICU 2. Neonatal ICU |
| Have you taken special training on ICU? | 1. yes  2. no |
| Height of respondent | _____________ |
| Weight of respondent | _____________ |
| Body mass index of respondents | ____________ |
| Have you a habit of doing regular exercise? | 1. yes 2. no |
| Have you ever smoked cigarette? | 1. yes 2. no |
| Have you ever used alcohol drinks? | 1. yes 2. no |
| Have you ever chewing Khat? | 1. yes 2. no |
| Have you rest interval after doing nursing procedures? | 1. yes 2. no |
| For how long did you sleep per day? | ___________hrs./day |

| This part assesses about job stress status. Each item is scored from 1(never) to 5(very often. Read each statement carefully and indicate how you feel about each statement. | | | | | |
| --- | --- | --- | --- | --- | --- |
|  | Never (1) | Rarely (2) | Sometimes (3) | Often (4) | Very often (5) |
| Conditions at work are unpleasant or sometimes even unsafe |  |  |  |  |  |
| I feel that my job is negatively affecting my physical or emotional well -being |  |  |  |  |  |
| I have too much work to do |  |  |  |  |  |
| I find it difficult to express my opinions or feelings about my job conditions to my superiors |  |  |  |  |  |
| I feel that job pressures interfere with my family or personal life |  |  |  |  |  |
| I have inadequate control or input over my work duties |  |  |  |  |  |
| I receive inappropriate recognition or rewards for good performance |  |  |  |  |  |
| I can’t able to utilize my skills and talents to the fullest extent at work? |  |  |  |  |  |
